# CD47 antibody inhibits tumor recurrence in a clinical relevant glioblastoma animal model

**DOI:** 10.1186/2051-1426-3-S2-P249

**Published:** 2015-11-04

**Authors:** Huaiyang Zhu, Ning Yang, Lina Leiss, Cecilie B Rygh, Per Ø Enger, Rolf Bjerkvig, Jian Wang

**Affiliations:** 1 University of Bergen, Bergen, Norway; 2 Qilu Hospital of Shandong University, Jinan, China

## 

Glioblastoma multiform (GMB), as a major unsolved clinical challenge, remains among the most aggressive malignan cies in human brain with poor prognosis. Although radiotherapy and chemotherapy that were given to patients after s urgery may improve overall survival, the treatment outcomes in general are poor. Thus, new therapeutic strategy for GBM is urgently needed. CD47, a tumor cell surface marker, plays as “don't eat me signal” through binding its recept or SIRPα on macrophages and the antibody against CD47, which blocks interactions of CD47 with SIRPα, has been shown to lead to tumor destruction1. Furthermore, CD47 is a prognostic marker as its expression predisposes cancer patients to a poorer survival outcome. This has significant clinical implications since approximately more than 80% of patients with the most GBMs, overexpress CD47. The purpose of the current study is to evaluate the feasibility of tar geting CD47 therapeutically as well as to explore its mechanism in a highly clinical relevant GBM nude rat model. In t he study, we have developed a novel xenograft surgical resection and tumor recurrence GBM model in parallel with t he patient settings in clinic. The model accounted for the therapeutic benefit of surgical resection and demonstrated t hat recurrent tumors higher proliferation and more dilated vessels in comparison with the primary tumors. It also has been shown that the tumor resection recruited more macrophages to the surgical site and by using the anti-CD47 antibody significantly prolonged animal survival. Moreover, angiogenesis array showed the angiogenic factors were down-regulated in the group of the animals treated with CD-47 antibody and cytokines array showed the cytokines that are related to immune responses and antitumor activities were up-regulated by the CD47 antibody. These results suggest that the presence of macrophages postoperatively was needed to mediate the anti-tumor effects of the anti-CD47 antibody and CD47 is the potential therapeutic target for recurrent GBM.

